# Enhanced metastatic potential in the MB49 urothelial carcinoma model

**DOI:** 10.1038/s41598-019-43641-5

**Published:** 2019-05-15

**Authors:** Yu-Ru Liu, Peng-Nien Yin, Christopher R. Silvers, Yi-Fen Lee

**Affiliations:** 1 Department of Urology, University of Rochester Medical Center, Rochester, NY 14642 USA; 2 Department of Pathology, University of Rochester Medical Center, Rochester, NY 14642 USA

**Keywords:** Bladder cancer, Bladder

## Abstract

Recent data suggest that patients with a basal/stem-like bladder cancer (BC) subtype tend to have metastatic disease, but this is unconfirmed. Here we report the identification of murine MB49 cell line sub-clones with stem-like characteristics in culture. Subcutaneous implantation of S2 and S4 MB49 sub-clones into immunocompetent mice resulted in lung metastases in 50% and 80% of mice respectively, whereas none of the mice implanted with the parental cells developed metastasis. Gene profiling of cells cultured from S2 and S4 primary and metastatic tumors revealed that a panel of genes with basal/stem-like/EMT properties is amplified during metastatic progression. Among them, *ITGB1*, *TWIST1* and *KRT6B* are consistently up-regulated in metastatic tumors of both MB49 sub-clones. To evaluate clinical relevance, we examined these genes in a human public dataset and found that *ITGB1* and *KRT6B* expression in BC patient tumor samples are positively correlated with tumor grade. Likewise, the expression levels of these three genes are correlated with worse clinical outcomes. This MB49 BC metastatic pre-clinical model provides a unique opportunity to validate and recapitulate results discovered in patient studies and to pursue future mechanistic therapeutic interventions for BC metastasis.

## Introduction

Bladder cancer (BC) is one of the most prevalent cancers in American men and is consistently among the six most common cancers in the United States, with approximately 429,800 new cases and 165,100 deaths annually worldwide^1^. Of all newly diagnosed cases of BC, 70–80% arise as non-muscle invasive (NMI) BC. These tumors often recur, and a subgroup of high-risk lesions frequently progress to muscle invasive (MI) BC. Conversely, 20–30% present as MIBC disease. Invasion of the muscular layer commonly portends progression to metastasis. In fact, 30–50% of MIBCs progress to metastasis despite radical cystectomy. Localized BCs have a favorable outcome, but regionally and distantly metastatic BCs have 5-year survival rates of only 34% and 5% respectively^2–4^. BC mortality is usually the result of metastatic progression^5^, yet there are currently few treatment options for metastatic BC. Systemic chemotherapy, the current standard, can seldom achieve durable disease control. BC often metastasizes to lymph nodes, bone, lung and peritoneum^6^, and the heterogeneous nature of BC leads to highly variable clinical outcomes and responses to therapy. Recent data obtained from whole genome/exome profiling of individual patient tumors revealed the molecular features and clinical implications of BC subtypes^7–9^. However, these data have not identified specific genetic alterations that drive disease progression, and these findings have not yet translated to evidence-based molecular cancer diagnostics and targeted therapies. Therefore, it is critical to understand the underlying biology and key molecular events that drive BC metastasis; however, research has been hampered by limitations of the preclinical animal models that recapitulate the pathological process of BC metastatic progression in humans^10,11^. MB49, one of the most-used murine bladder carcinoma cell lines, was generated by exposing C57BL/6 primary bladder epithelial cell explants to 7,12-dimethylbenz[α]anthracene (DMBA) for 24 hours followed by long-term culture^12^. It shares several pivotal tumor characteristics with human BC, such as cell surface markers, sensitivity to apoptosis, and immunological profile^13–16^. However, MB49 isograft tumors grow rapidly in mice, and in many instances, the mice need to be sacrificed before metastasis occurs. A more aggressive MB49 variant, MB49-I, was developed through 13 serial passages of subcutaneous MB49 tumors. MB49-I BC cells displayed aggressively invasive behavior *in vitro* and *in vivo*, and such invasiveness is probably due to elevated expression levels of decorin (*DCN*)^17^, yet its relevance to human BC has not yet been confirmed.

Using recent data obtained from the whole genome sequencing and transcriptome profiling, BCs have been grouped into basal and luminal molecular subtypes that possess distinct biological and clinical features^7,18,19^. Basal cells express biomarkers characteristic of cancer stem cells (CSC) and epithelial-to-mesenchymal transition (EMT) that are implicated in BC metastasis^7^. Patients with the basal/CSC BC subtype tend to have cancers of more advanced stage and to have higher likelihood of metastasis. Similarly, basal/CSC human BC orthotopic xenografts in mice are also more metastatic than luminal/epithelial cells^20^. These reports support the functional role of CSCs in metastasis of BC.

To recapitulate these key metastatic molecular features presented in the clinical scenario, we report the establishment of pro-metastatic MB49 sub-clones which form spheroidal 3D structures in culture. Tumor-derived spheroids have been reported to be enriched with CSCs or cells with stem-like properties^21^. We have characterized the behavior of the MB49 sub-clones *in vitro* and *in vivo*, and molecular profiling of cells derived from primary tumors *vs*. metastatic tumors suggests a panel of differentially expressed CSC- and EMT-related genes in primary tumors that can be predictive of their metastatic potential.

## Results

### Isolation and characterization of stem-like MB49 sub-clones

**Figure 1:**
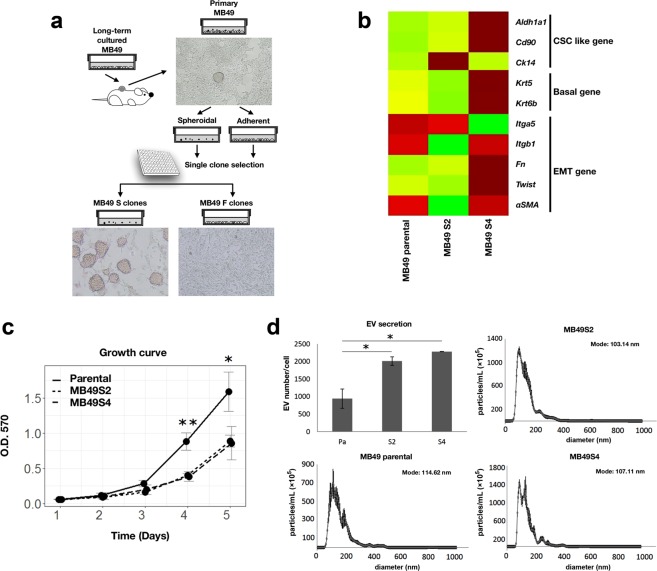
Metastasis-prone MB49 sub-clone selection and basic cellular characters. (**a**) Diagram illustrating the generation of spheroid-forming MB49 clones (S) and adherent MB49 clones (F). (**b**) Heat map illustrating the expression profile of CSC, EMT and basal genes in MB49 parental, MB49S2 and MB49S4. (**c**) *In vitro* proliferation of MB49 parental and sub-clones as measured by the MTT assay. (**d**) EV secretion rates and EV size distributions of MB49 parental and sub-clones *in vitro* as determined by NTA.

The MB49 syngeneic murine model of BC has been widely used for more than 35 years^12,22^. Within a tumor mass composed of billions of cells, usually only a small percentage of cells are able to metastasize. Cancer cells are generally cultured *in vitro* using media which support the growth of differentiated and less tumorigenic cells, resulting in some loss of *in vivo* tumorigenicity^23^. To select a small subpopulation of adaptable cancer cells that are highly tumorigenic, we implanted long-term cultured MB49 cells into C57BL/6 mice subcutaneously, which allowed predominant growth of adaptable MB49 cells *in vivo*. Primary subcutaneous MB49 tumors were resected, mechanically disrupted and trypsin digested to obtain single cells. As illustrated in Fig. 1a, the cells were a heterogeneous mix of mostly adherent cells and a small set of cells that formed spheroidal 3D structures in culture. We hypothesized that the MB49 cell subpopulation found in the spheroids would be stem-like, basal, and highly metastatic as compared to the adherent cells. Single cell clones were established through serial dilution of cells dissociated from the spheroids or the cells in the adherent subpopulation. We initially selected five spheroidal and three adherent single cell sub-clones named S1-5 and F1-3 respectively. The morphology and spheroid-generating character of these sub-clones appeared to be stable in culture for at least two months.

To assess the metastatic potential of the sub-clones, we conducted a pilot test by subcutaneous implantation into male C57BL/6 mice. Tumors developed to their allowable endpoints or until three weeks had elapsed, and then mice were sacrificed to histologically assess the formation of lung metastases. Three out of four spheroid-forming (S) sub-clones had developed lung metastases, whereas no metastases were found in MB49 parental or F sub-clone implanted animals (Supplementary Table 1). Among the metastatic sub-clones, subcutaneous S1 tumors grew rapidly and led to tumor excoriation within 10 days. One of the necessary features of a usable syngeneic metastasis model is a moderate growth rate that allows significant metastatic outgrowth to occur before the primary graft exceeds the acceptable tumor endpoint. For this reason, we chose the S2 and S4 spheroidal sub-clones for further *in vitro* and *in vivo* characterization due to their moderate tumor growth rates. Consistent with their stem-like morphology, we showed that S2 cells are enriched in CSC marker *CK14*, and S4 cells are enriched in CSC markers *ALDH1A1* and *CD90* and as well as basal markers *KRT5* and *KRT6B*, as compared to MB49 parental cells (Fig. 1b). Interestingly, the EMT markers *αSMA*, *ITGB1*, fibronectin (*FN1*) and *TWIST1* are elevated in S4 cells, but not in S2 cells. The S2 and S4 MB49 sub-clones also grow significantly more slowly than MB49 parental cells (Fig. 1c).

It has been reported that cancer cells produce more extracellular vesicles (EVs) than normal cells^24^, and tumor-derived EVs (TEVs) play essential roles in both primary tumor growth and metastatic outgrowth. TEVs can transfer cargo molecules to the local tumor microenvironment and facilitate tumor growth, and TEVs can also travel a distance and reprogram recipient stromal cells to support pre-metastatic niche formation and subsequently facilitate metastasis^25^. To examine whether the MB49 sub-clones release EVs at different rates and whether EV production is positively correlated with metastatic potential, we isolated EVs from conditioned media as described in our previous publications^26–28^ and measured particle size and concentration using nanoparticle tracking analysis (NTA). As expected, we found that both S2 and S4 MB49 sub-clones release significantly more EVs than parental MB49 cells, but there is no difference in EV size distribution (approximately 103–114 nm) among the three MB49 lines (Fig. 1d).

### MB49 S2 and S4 sub-clones develop lung metastases *in vivo*

**Figure 2:**
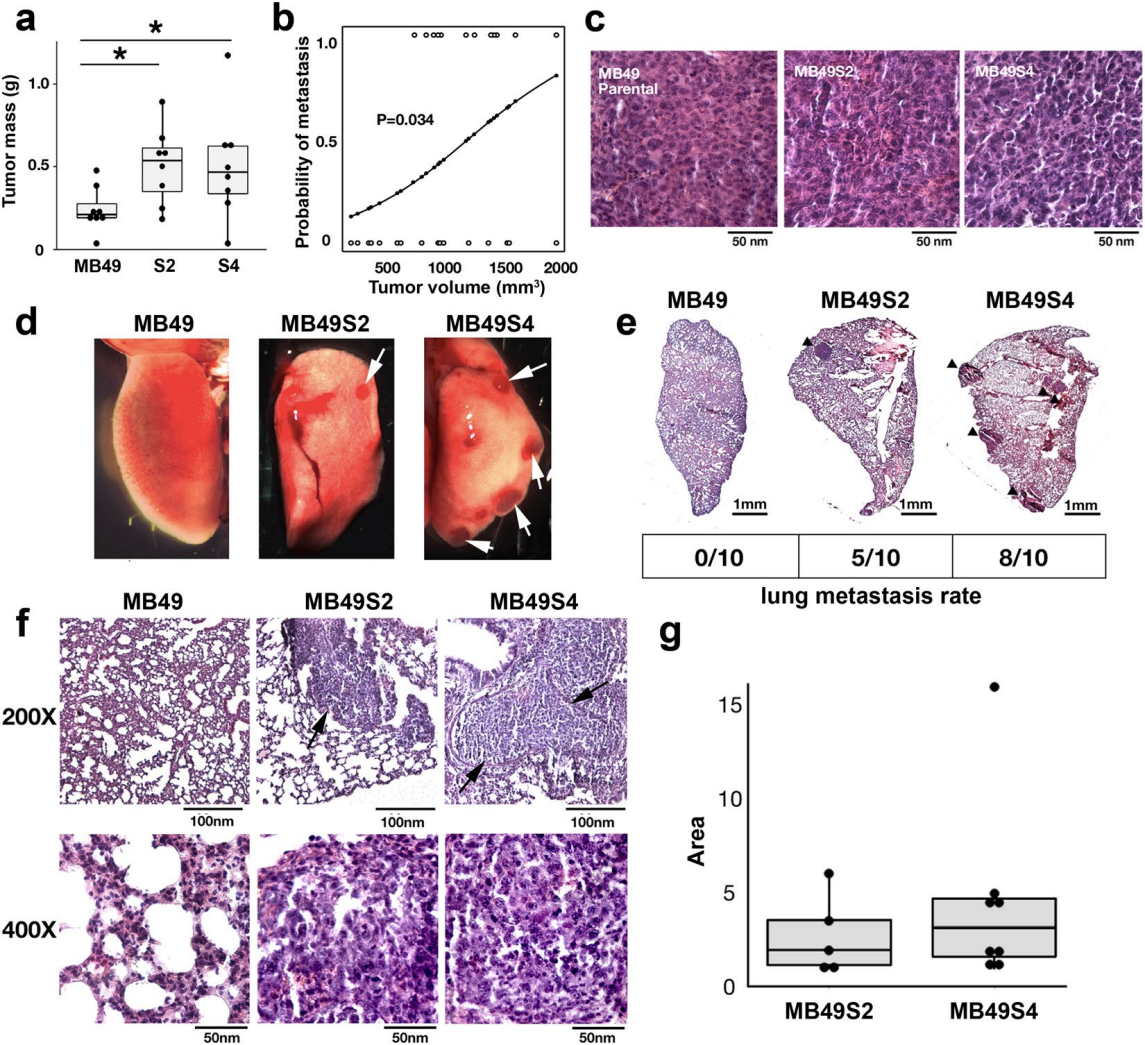
Lung metastasis in mice with subcutaneous MB49 sub-clone isografts. (**a**) Masses of the subcutaneous primary tumors collected on day 19. Asterisks represent significant difference (p value < 0.05, Student’s *t*-test). (**b**) Logistic regression showing correlation between tumor volume and the incidence of metastasis. (z score = 2.121, p-value = 0.034). (**c**) H&E staining of subcutaneous primary tumors. (**d**) Representative photographs of lung lobes 19 days after subcutaneous implantation of MB49 parental cells or S2 and S4 sub-clone cells. White arrows indicate grossly visible lung lesions. (**e**) Composite photomicrograph showing H&E staining of whole mount lung lobe. Urothelial carcinoma lesions are indicated by black arrows. The rates of lung metastasis at days 19–22 are given as the metastasis-positive animal number/total animal number. (**f**) H&E staining of lungs shown at 200x and 400x magnification. (**g**) Metastatic lesion area in both MB49S2 and MB49S4 metastasis-positive left lungs as measured using NIH ImageJ.

To fully assess their metastatic potential *in vivo*, we implanted both parental and spheroid-forming MB49 S2 and S4 sub-clones subcutaneously into ten male C57BL/6 mice per group. As expected, all tumors grew rapidly *in situ*, with MB49 parental, S2 and S4 cells forming palpable primary tumors within 7 days. All tumors remained within allowable endpoints until day 19, when eight mice per group were sacrificed for analysis of both subcutaneous tumors and lung metastatic burden. In contrast to the *in vitro* cell growth rates, S2 and S4 sub-clones formed larger primary tumors than parental MB49 cells by day 19 (Fig. 2a), and logistic regression analysis showed that the primary tumor volume was positively correlated to the probability of lung metastasis (Fig. 2b, *p* = 0.034). Hematoxylin and eosin (H&E) staining of the primary tumors confirmed the presence of high-grade urothelial carcinoma (Fig. 2c). We found no morphological differences among the primary tumors of the three MB49 lines.

The remaining mice were sacrificed on day 21, and all lungs were examined for the presence of urothelial carcinoma. We found that 4/10 of the S2 and 7/10 of the S4-implanted mice had developed grossly visible lung lesions (Fig. 2d); in contrast, no lung lesions were seen in the parental MB49-implanted mice. Histological examination found that 5/10 of the S2 and 8/10 of the S4-implanted mice had metastatic high-grade urothelial carcinoma in their lungs, and no metastatic tumors were found in more than 900 lung sections histologically examined from the 10 animals implanted with MB49 parental cells (Fig. 2e). The histology of lung metastases in the S2- and S4-implanted groups (Fig. 2e,f) was similar to that of the primary tumors. We further compared the severity of metastases from S2 and S4 sub-clone implanted animals. There was no significant difference in the area (Fig. 2g) or number (Table 1) of metastatic lung lesions between the S2 and S4 implanted groups.

**Table 1:** Incidence of MB49 sub-line lung metastasis.

MB49 line	Mice with lung metastases	Avg. tumors per lung section
P	0/10	0
S2	5/10	1.2
S4	8/10	1.8

The lung metastasis incidence of three MB49 cell lines, parental (P), S2 and S4, was measured 19–22 days following subcutaneous implantation into C57BL/6 mice.

In summary, we have isolated MB49 sub-clones with spheroid-forming ability which develop lung metastases from subcutaneously engrafted tumors, and while S4 sub-clones have a higher metastatic rate than S2, there were no differences in lung metastatic lesion size or frequency between the two groups.

### Elevated expression of CSC and EMT related genes metastatic sub-clones *in vivo*

**Figure 3:**
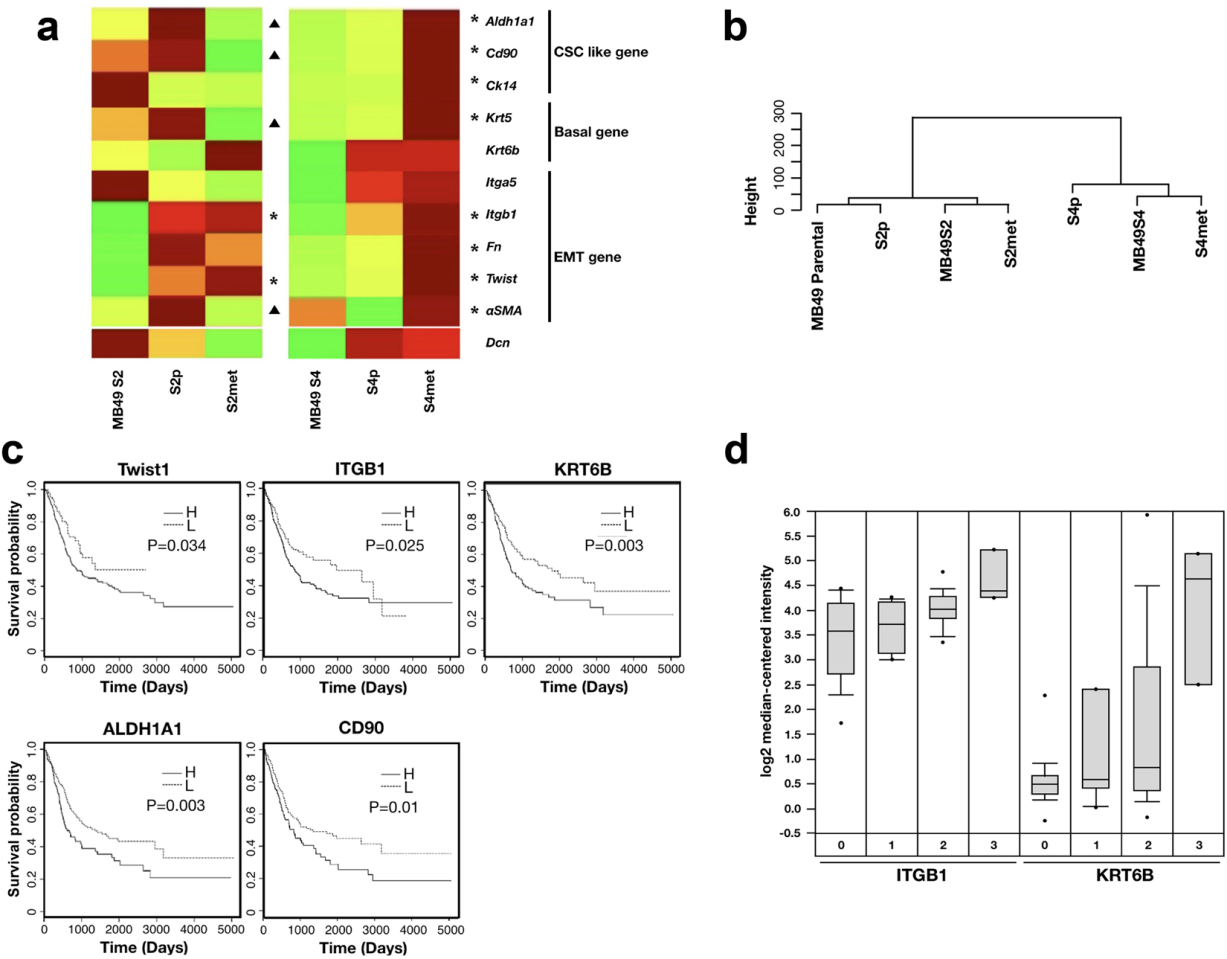
Identification of highly-expressed CSC genes in MB49S2 and MB49S4. (**a**) Heat map illustrating the expression profile of stem-like, EMT and basal genes. ▲ marks genes that have expression elevated in primary tumors and reduced in the lung metastases. * marks genes that have expression elevated in lung metastases. (**b**) Cluster dendrogram of MB49 parental and sub-clones. (**c**) Kaplan-Meier survival curves of human patients with high (H) or low (L) bladder tumor tissue expression of key genes highly expressed in the metastatic MB49 sub-clones. Survival and gene expression data were withdrawn from the Human Protein Atlas. (**d**) Oligonucleotide array expression of *ITGB1* and *KRT6B* mRNA in bladder cancer tissues (0: No value; 1: Grade 2, 2: Grade 3, 3: Grade 4; original data as published in Dyrskjøt, *et al*.^30^). The p-values of infiltrating bladder urothelial carcinoma vs. normal are 0.00079 for *ITGB1* and 0.002 for *KRT6B*.

To understand the molecular pathways involved in BC metastatic progression, we resected tumors from S2 and S4 MB49 sub-clone engrafted mice and isolated cells from both the primary grafts (S2p, S4p) and lung metastatic tumors (S2met, S4met). The molecular profiles of the key pathways involved in BC metastasis, including CSC, EMT, and basal subtypes, were compared among the initial lines and the re-derived cells by quantitative RT-PCR. We found the S4p and S4met cells share a similar pattern in which nine genes were up-regulated during the progression from *in vitro* culture to primary graft and finally lung metastasis (Fig. 3a). High expression levels were detected in certain genes that are characterized as stem-like (*ALDH1A1, CD90* and *CK14*), belonging to the basal subtype (*KRT5* and *KRT6B*) and associated with EMT (*ITGA5, ITGB1, FN1, ACTA2* and *TWIST1*). These data agree with results from The Cancer Genome Atlas (TCGA) project, in which BC patients expressing basal, CSC and EMT biomarkers are found to have cancers of more advanced stage and to have higher likelihood of metastasis. Interestingly, the MB49S2 series, morphologically indistinguishable from S4, showed a mixed molecular alteration during development of primary subcutaneous tumors to lung metastasis. Three genes were up-regulated in lung metastases (Fig. 3a, marked *), while 4 genes upregulated in the primary grafts were again downregulated in metastatic tumors (Fig. 3a, marked ▲). Based on their gene expression levels, we performed cluster dendrogram analysis to assess the hierarchical relationship between the S2 and S4 series, finding that the less aggressive S2 sub-clones (MB49S2, S2p, S2met) were clustered with the non-metastatic MB49 parental cells (Fig. 3b).

The three genes (*TWIST1*, *ITGB1* and *KRT6B*) consistently up-regulated in S2met and S4met are particularly interesting for their potential as BC metastasis biomarkers. To verify their clinical relevance, we examined whether gene expression can predict disease progression using human sample data retrieved from the Human Protein Atlas^29^ (www.proteinatlas.org). As shown in Fig. 3c, we found expression levels of *TWIST1*, *ITGB1* and *KRT6B* are correlated with worse prognosis in patients with BC. Furthermore, mRNA expression levels of *ITGB1* and *KRT6B* in a published microarray dataset^30^ were significantly higher in infiltrating bladder urothelial carcinoma patient tissue samples vs. normal controls (Fig. 3d). In summary, we have identified a panel of genes with basal/stem-like/EMT properties that are selectively amplified during metastatic progression in the murine MB49 cell syngeneic model.

## Discussion

Animal models are invaluable for investigating the complex, multiple-step metastasis process that enables the growth of cancer cells detached from primary tumors, intravasation, migration, extravasation, and finally colonization of distant organs. The availability of preclinical BC animal models, especially metastatic BC models, is limited. Few genetically engineered mouse BC models display invasive or metastatic phenotypes^10^, and both engrafted and carcinogen-induced orthotopic BC model mice often die before metastasis develops^31^. To acquire more aggressive BC cells for *in vivo* studies, researchers have applied serial passaging through animals. For instance, tail vein injection of Lu11/2 cells produced by continued passaging of subcutaneous primary UMUC3 cells leads to lung metastasis in immunocompromised mice^32^, and a similar approach was used in improving the metastatic potential of the T24 variant, T24T^33^. While the immune system plays important roles in cancer metastasis, the use of immunocompromised mice in xenograft models limits the investigation of interactions between the tumor and its host immune response. Among all BC models, an orthotopic model that closely mimics the tumor microenvironment in humans is ideal. However, this model also has limitations and challenges. Studies using transurethral instillation of cancer cells and treatment agents traditionally use female animals, as catheterization of the male urethra is extremely difficult^34^. Furthermore, instilled cancer cells do not generally adhere to the unmodified bladder wall, and it is necessary to damage the urothelium to facilitate cell adhesion^22^. Various methods have been used to create these injuries and often result in inconsistent tumor uptake rates^22^. In some cases, the wounded urothelium can cause the rapid invasion of implanted BC cells into underlying layers, and this invasion behavior does not reflect their aggressiveness^35,36^. Thus, one needs to be cautious in interpreting results generated from orthotopic BC models.

In the current study, we applied the MB49 syngeneic metastatic model to examine the progression of MB49 sub-clones from primary subcutaneous tumors to lung metastases in immunocompetent C57BL/6 host mice. The subcutaneous tumor model is one of the most widely used cell-based engraftment models and has considerable advantages. It is technically simple, since tumors can be implanted in animals of both sexes, and also highly reproducible, allowing easy access to primary tumors for analyses of the functional relevance of candidate genes. This MB49 syngeneic metastatic model preserves the context of the intact immune system; a disadvantage of this model, however, is that the tumor microenvironment (host stroma and extracellular matrix) found in the subcutaneous implantation site might not completely mirror that found in the bladder.

The conception of the CSC as the origin of cancer was proposed over four decades ago. CSCs compose a small subpopulation of cancer cells that share characteristics similar to embryonic stem cells. CSCs possess self-renewal potential through activation of several embryonic stem cell genes that are silenced in fully differentiated cells. In addition, CSCs can also be transformed from the mutation of resident normal stem cells and/or from dedifferentiation of non-stem cancer cells (non-CSCs) via cross-talk with the tumor microenvironment. The bidirectional conversion between CSCs and non-CSCs was reported in numerous studies^37,38^. In the current study, we showed that a CSC-like subpopulation of MB49 cells was produced by *in vivo* selection in the immunocompetent host environment, and this subpopulation was proven to be pro-metastatic. Similarly, it has been reported that the expression of *Snail* and *Twist* in breast cancer cells can induce EMT and a stem cell-like character that would lead to increased metastatic potential^39–41^. Our data support the involvement of the CSC and EMT pathways in mediating BC metastasis.

Interestingly, we found that two EMT genes activated by the Wnt signaling pathway, *TWIST1* and *ITGB1*, are induced in the lung metastatic cells, S2met and S4met. This suggests that the tumor microenvironment participates in tumor evolution during the course of disease progression and is associated with activation of Wnt signaling, which is known to be activated and involved in BC progression^38,42^. The *TWIST1* and *ITGB1* genes are Wnt signaling downstream targets and promote EMT^43^. Recently, exosomes were found to be vehicles for transporting active Wnt ligands^44^. Therefore, it would be of interest to examine if EVs released from S2 and S4 cells contain Wnt ligands that activate EMT signals in an autocrine mechanism. In addition, we found an elevated expression of *KRT6B*, a member of the extracellular matrix protein family known to interact with Notch1 and contribute to renal carcinoma progression^45^. Another gene of interest is decorin, which was previously found to be highly expressed in another metastatic MB49 sub-line, MB49-I^17^, but we found it to be elevated only in S2met and not S4met cells. The roles of decorin in metastasis remain controversial, as several studies suggest that it functions as a tumor suppressor^46–48^ while others suggest that it promotes tumor invasiveness, metastasis, and angiogenesis^49–53^. More research is needed to further delineate decorin’s functions.

In summary, we have demonstrated a novel syngeneic metastatic BC model with high reproducibility and clinical relevance. This model provides a unique opportunity to pursue evidence-based translational research for future mechanistic clinical therapeutic interventions to overcome BC metastasis.

## Methods

### Cell culture and establishment of primary cells

The parental MB49 cells were a gift from Dr. Timothy Ratliff at Purdue University College of Veterinary Medicine and cultured in RPMI 1640 containing 10% fetal bovine serum (FBS). Cells were isolated from primary and metastatic MB49 tumors using the ATCC® Primary Cell Culture Guide. Tumors were resected from the primary subcutaneous injection site and from the lung, dissected into small pieces approximately 2 mm in diameter, and then incubated with 0.25% trypsin-EDTA to further dissociate into single cells. Cell suspensions were passed through 70 µm filters to remove debris and washed with culture medium. The yield and viability were determined by a trypan blue exclusion method (Vi-CELL XR Cell Counter, Beckman) and the single cells were plated and cultured in RPMI with 10% FBS. Cultures which spontaneously formed spheroids were maintained by collecting both adherent cells (using 0.25% trypsin-EDTA) and spheroids, disaggregating the spheroids using a 10 minute incubation in 0.25% trypsin-EDTA followed by repeated pipetting, and sub-culture of the resulting cell mixture.

### Proliferation assay

Cell growth was determined using methylthiazolyldiphenyl-tetrazolium bromide (MTT). Briefly, MB49 parental cells and early passage (5–10) S2 and S4 sub-clones were used. 7 × 10^3^ cells were seeded into 12-well plates with 0.5 ml of medium per well. At harvest time points, MTT solution (1.25 mg/ml in RPMI) was added to cultures at a 1:1 volume ratio and incubated for two hours at 37 °C. Spheroids were collected using a brief centrifugation and returned to the culture vessels prior to DMSO extraction of the Formazan crystals. Absorbance of the formazan solution was read spectrophotometrically at 570 nm.

### EV isolation and nanoparticle tracking analysis

Cell culture supernatants were processed immediately after collection by serial centrifugation at 400 × g for 10 minutes and 15500 × g for 30 minutes to remove cells and debris and then stored at −80 °C. EVs were isolated from thawed samples by ultracentrifugation performed twice at 200,000 × g for 70 minutes at 4 °C, and the resulting pellets were re-suspended in a small volume of DPBS. Aggregates were removed from the samples by another 15500 × g centrifugation for 5 minutes. Final total protein concentrations of the samples were measured by Micro BCA assay (Thermo Fisher Scientific, #23235), and samples were stored at −80 °C. Particle size distribution and concentration in EV isolates were measured using a NanoSight NS300 (Malvern Instruments). Each sample was diluted 1:1000 in DPBS with negligible background signal and recorded into five video files of 30 seconds each.

### *In vivo* mouse model

The study was approved by the University of Rochester Committee on Animal Resources under protocol #2013-025, and the mice were kept in a specific pathogen-free environment at the animal facility of the University of Rochester Medical Center. All experiments were performed in accordance with relevant guidelines and regulations. MB49 parental/S2/S4 cells at 70–80% confluence (in passage 5–7), were detached, washed twice with PBS, and re-suspended to make ten million cells per ml in RPMI with 10% FBS medium and left on ice no longer than one hour. 8 week old male C57BL/6 mice were purchased from Jackson Laboratory and allowed 1–2 weeks of acclimatization in the vivarium after arrival. Animals were anaesthetized by isoflurane, and cells were injected into the flank of each mouse subcutaneously (1 × 10^6^ cells in 100 µL). Tumor length and width were measured using calipers, and tumor volume was determined using the ellipsoid formula.

### Total RNA extraction and quantitative real-time PCR

Total RNA was collected from cells using acid guanidinium thiocyanate-phenol-chloroform extraction and quantified using spectrophotometry (NanoDrop, Thermo Fisher Scientific, Waltham, MA). First strand cDNA was synthesized using 1 μg total RNA in a 20 μL reaction using the iScript cDNA synthesis kit instructions (Bio-Rad, Hercules, CA). cDNA levels were measured in triplicate by iQ SYBR Green (Bio-Rad), and relative target expression was normalized to *GOT1*, *GAPDH* and *HPRT*. Primer sequences are listed in Supplementary Table 2.

### Histological examination of tumors and pathology assessment

The left lung lobe was collected from each animal and fixed in 10 mL of 4% paraformaldehyde for at least 48 hours before paraffin processing. Embedded specimen blocks were cut into 10 µm thick sections, and all of the sectioned tissues were collected on frosted glass slides. H&E staining was performed on all slides, and slides were photographed using a Leica DM5000 B microscope. The area of the metastatic lung lesions was calculated in five randomly selected metastasis-positive sections using NIH ImageJ^54^. The tumor grading was performed by licensed pathologists, Dr. Hiroshi Miyamoto and Dr. Zhiming Yang.

### Statistical analysis

All of the statistical analysis was conducted using the R software environment (R Foundation for Statistical Computing). One-way ANOVA with Scheffe post-testing was used to compare results among different groups. The logistic regression was calculated using the Pearson Chi-square test. Cell proliferation was plotted with the general plot function and ggplot2 with the geom_line function. The gene heatmap was plotted using the heatmap function. The cluster analysis was conducted by using Ward’s hierarchical agglomerative clustering method. Survival data and gene expression values (fragments per kilobase per million mapped fragments, FPKM) were withdrawn from the Human Protein Atlas for use in the Kaplan-Meier plots

## Supplementary information

Supplementary Information

## References

[1] Torre LA (2015). Global cancer statistics, 2012. CA: a cancer journal for clinicians.

[2] Steinberg GD, Trump DL, Cummings KB (1992). Metastatic bladder cancer. Natural history, clinical course, and consideration for treatment. The Urologic clinics of North America.

[3] Chang SS, Cookson MS (2005). Radical cystectomy for bladder cancer: the case for early intervention. The Urologic clinics of North America.

[4] Cancer Facts and Figures. (American cancer Society, 2017).

[5] von der Maase H (2000). Gemcitabine and cisplatin versus methotrexate, vinblastine, doxorubicin, and cisplatin in advanced or metastatic bladder cancer: results of a large, randomized, multinational, multicenter, phase III study. Journal of clinical oncology: official journal of the American Society of Clinical Oncology.

[6] Shinagare AB (2011). Metastatic pattern of bladder cancer: correlation with the characteristics of the primary tumor. AJR. American journal of roentgenology.

[7] Choi W (2014). Identification of distinct basal and luminal subtypes of muscle-invasive bladder cancer with different sensitivities to frontline chemotherapy. Cancer Cell.

[8] Robertson AG (2017). Comprehensive Molecular Characterization of Muscle-Invasive Bladder. Cancer. Cell.

[9] Robertson AG (2018). Comprehensive Molecular Characterization of Muscle-Invasive Bladder. Cancer. Cell.

[10] Kobayashi T, Owczarek TB, McKiernan JM, Abate-Shen C (2015). Modelling bladder cancer in mice: opportunities and challenges. Nature reviews. Cancer.

[11] John BA, Said N (2017). Insights from animal models of bladder cancer: recent advances, challenges, and opportunities. Oncotarget.

[12] Summerhayes IC, Franks LM (1979). Effects of donor age on neoplastic transformation of adult mouse bladder epithelium *in vitro*. Journal of the National Cancer Institute.

[13] Loskog A, Totterman TH, Bohle A, Brandau S (2002). *In vitro* activation of cancer patient-derived dendritic cells by tumor cells genetically modified to express CD154. Cancer gene therapy.

[14] Yang AS, Lattime EC (2003). Tumor-induced interleukin 10 suppresses the ability of splenic dendritic cells to stimulate CD4 and CD8 T-cell responses. Cancer research.

[15] O’Donnell MA (2004). The essential role of interferon-gamma during interleukin-12 therapy for murine transitional cell carcinoma of the bladder. The Journal of urology.

[16] Loskog A (2004). Adenovirus CD40 ligand gene therapy counteracts immune escape mechanisms in the tumor Microenvironment. Journal of immunology.

[17] El Behi M (2013). An essential role for decorin in bladder cancer invasiveness. EMBO molecular medicine.

[18] Atlas Research N (2014). Comprehensive molecular profiling of lung adenocarcinoma. Nature.

[19] Damrauer JS (2014). Intrinsic subtypes of high-grade bladder cancer reflect the hallmarks of breast cancer biology. Proceedings of the National Academy of Sciences of the United States of America.

[20] McConkey DJ, Choi W, Ochoa A, Dinney CPN (2016). Intrinsic subtypes and bladder cancer metastasis. Asian journal of urology.

[21] Weiswald LB, Bellet D, Dangles-Marie V (2015). Spherical cancer models in tumor biology. Neoplasia.

[22] Loskog A (2005). Optimization of the MB49 mouse bladder cancer model for adenoviral gene therapy. Laboratory animals.

[23] Mitra A, Mishra L, Li SL (2013). Technologies for deriving primary tumor cells for use in personalized cancer therapy. Trends Biotechnol.

[24] Kalluri R (2016). The biology and function of exosomes in cancer. J Clin Invest.

[25] Liu Y, Cao X (2016). Characteristics and Significance of the Pre-metastatic Niche. Cancer cell.

[26] Silvers CR (2016). Identification of extracellular vesicle-borne periostin as a feature of muscle-invasive bladder cancer. Oncotarget.

[27] Silvers CR, Miyamoto H, Messing EM, Netto GJ, Lee YF (2017). Characterization of urinary extracellular vesicle proteins in muscle-invasive bladder cancer. Oncotarget.

[28] Wu, C. H., Silvers, C. R., Messing, E. M. & Lee, Y. F. Bladder cancer extracellular vesicles drive tumorigenesis by inducing the unfolded protein response in endoplasmic reticulum of non-malignant cells. The Journal of biological chemistry, 10.1074/jbc.RA118.006682 (2018). 10.1074/jbc.RA118.006682 PMC6398136 30593508

[29] Uhlen, M. *et al*. A pathology atlas of the human cancer transcriptome. *Science (New York, N.Y.)* **357**, 10.1126/science.aan2507 (2017). 10.1126/science.aan2507 28818916

[30] Dyrskjot L (2004). Gene expression in the urinary bladder: a common carcinoma *in situ* gene expression signature exists disregarding histopathological classification. Cancer Res.

[31] Zuiverloon TCM, de Jong FC, Costello JC, Theodorescu D (2018). Systematic Review: Characteristics and Preclinical Uses of Bladder Cancer Cell Lines. Bladder cancer.

[32] Overdevest JB (2011). CD24 offers a therapeutic target for control of bladder cancer metastasis based on a requirement for lung colonization. Cancer research.

[33] Nicholson BE (2004). Profiling the evolution of human metastatic bladder cancer. Cancer research.

[34] Thai, K. H., Thathireddy, A. & Hsieh, M. H. Transurethral induction of mouse urinary tract infection. Journal of visualized experiments: JoVE, 10.3791/2070 (2010). 10.3791/2070 PMC3156012 20729806

[35] Gabriel U, Bolenz C, Michel MS (2007). Experimental models for therapeutic studies of transitional cell carcinoma. Anticancer research.

[36] Ding J (2014). Current animal models of bladder cancer: Awareness of translatability (Review). Experimental and therapeutic medicine.

[37] Marjanovic ND, Weinberg RA, Chaffer CL (2013). Cell plasticity and heterogeneity in cancer. Clinical chemistry.

[38] Urakami S (2006). Epigenetic inactivation of Wnt inhibitory factor-1 plays an important role in bladder cancer through aberrant canonical Wnt/beta-catenin signaling pathway. Clinical cancer research: an official journal of the American Association for Cancer Research.

[39] Morel AP (2008). Generation of breast cancer stem cells through epithelial-mesenchymal transition. Plos One.

[40] Mani SA (2008). The epithelial-mesenchymal transition generates cells with properties of stem cells. Cell.

[41] Kalluri R, Weinberg RA (2009). The basics of epithelial-mesenchymal transition. The Journal of clinical investigation.

[42] He X (2009). Differentiation of a highly tumorigenic basal cell compartment in urothelial carcinoma. Stem cells.

[43] Karreth F, Tuveson DA (2004). Twist induces an epithelial-mesenchymal transition to facilitate tumor metastasis. Cancer biology & therapy.

[44] Gross JC, Chaudhary V, Bartscherer K, Boutros M (2012). Active Wnt proteins are secreted on exosomes. Nature cell biology.

[45] Hu J, Zhang LC, Song X, Lu JR, Jin Z (2015). KRT6 interacting with notch1 contributes to progression of renal cell carcinoma, and aliskiren inhibits renal carcinoma cell lines proliferation *in vitro*. International journal of clinical and experimental pathology.

[46] Csordas G (2000). Sustained down-regulation of the epidermal growth factor receptor by decorin. A mechanism for controlling tumor growth *in vivo*. The Journal of biological chemistry.

[47] Iozzo RV, Moscatello DK, McQuillan DJ, Eichstetter I (1999). Decorin is a biological ligand for the epidermal growth factor receptor. Journal of Biological Chemistry.

[48] Santra M, Eichstetter I, Iozzo RV (2000). An anti-oncogenic role for decorin - Down-regulation of ErbB2 leads to growth suppression and cytodifferentiation of mammary carcinoma cells. Journal of Biological Chemistry.

[49] Benet M (2012). Wild type N-ras displays anti-malignant properties, in part by downregulating decorin. J Cell Physiol.

[50] Cawthorn TR (2012). Proteomic Analyses Reveal High Expression of Decorin and Endoplasmin (HSP90B1) Are Associated with Breast Cancer Metastasis and Decreased Survival. Plos One.

[51] Dil, N. & Banerjee, A. G. A role for aberrantly expressed nuclear localized decorin in migration and invasion of dysplastic and malignant oral epithelial cells. *Head Neck Oncol* **3**, 10.1186/1758-3284-3-44 (2011). 10.1186/1758-3284-3-44 PMC3198745 21958730

[52] Fiedler LR, Eble JA (2009). Decorin regulates endothelial cell-matrix interactions during angiogenesis. Cell Adhes Migr.

[53] Zafiropoulos A (2008). Decorin-induced growth inhibition is overcome through protracted expression and activation of epidermal growth factor receptors in osteosarcoma cells. Mol Cancer Res.

[54] Schneider CA, Rasband WS, Eliceiri KW (2012). NIH Image to ImageJ: 25 years of image analysis. Nature methods.

