# Supplementary material for: Enhanced metastatic potential in the MB49 urothelial carcinoma model
Source: Sci Rep. 2019 May 15;9:7425. doi: 10.1038/s41598-019-43641-5 (PMC6520404; doi:10.1038/s41598-019-43641-5)
Supplement: Supplementary file 1 — Supplementary Information [file 41598_2019_43641_MOESM1_ESM.pdf]

Supplementary Information

Enhanced metastatic potential in the MB49 urothelial carcinoma model

Yu-Ru Liu[1], Peng-Nien Yin[1], Christopher R. Silvers[1], Yi-Fen Lee*[1,2]

Departments of [1]Urology and [2]Pathology, University of Rochester Medical Center, Rochester NY, 14642, USA

Corresponding Author: Yi-Fen Lee, Department of Urology, University of Rochester Medical Center, 601 Elmwood Ave, Box 656, Rochester, NY 14642. Phone: (585) 275-9702. E-mail: yifen_lee@urmc.rochester.edu

[+]The first two authors contributed equally to this work.

Supplementary Table 1. Incidence of lung metastasis in a pilot test of MB49 sub-clones.

MB49 line	mice with lung metastases
parental	0/2
F1	0/3
F2	0/3
F3	0/3
S1	3/3
S2	1/2
S3	0/4
S4	1/4
S5	1/6

The lung metastasis incidence of eight candidate MB49 sub-clones was measured 10-21 days following subcutaneous implantation into C57BL/6 mice.

Supplementary Table 2. Primer sequences used in qPCR.

	Gene name	Forward	Reverse
	**DCN**	TTCCTACTCGGCTGTGAGTC	AAGTTGAATGGCAGAACGC
**Basal marker genes**	**CD44**	TTTGCATTGCAGTCAACAGTC	GTTACACCCCAATCTTCATGTCCAC
	**KRT5**	TCTGCCATCACCCCATCTGT	CCTCCGCCAGAACTGTAGGA
	**KRT6B**	GCTCAAACATAGGCTCCAGGTT	CCATCAAGAGTCAAACTAGCCAC
	**KRT14**	AGCGGCAAGAGTGAGATTTCT	CCTCCAGGTTATTCTCCAGGG
**CSC marker genes**	**ALDH1A1**	TGATGTTGTCAGCCCAGTGC	TTGCGACTGTCTTGAGC
	**CD49**	TCTATTCCTGTTGCCGTCACAA	CGATGACAGCATTCCCCGATA
	**CK14**	CGGCCCACTGAGATCAAAGAC	AAATGCCAAGCCCCATTTTAGG
	**CD90**	CTGCAGCTAGGGGAGTCCAG	GATGACCCCTAATGTCCAGGTC
**EMT marker genes**	**ITGA5**	CTTCTCCGTGGAGTTTTACCG	GCTGTCAAATTGAATGGTGGTG
	**ITGB1**	ATGCCAAATCTTGCGGAGAAT	TTTGCTGAGATTGGTGACATT
	**ACTA2 (αSMA)**	GTCCCAGACATCAGGGAGTAA	TCGGATACTTCAGAGTCAGGA
	**FN1**	GCTCAGCAAATCGTGCAGC	CTAGGTAGGTCCGTTCCCACT
	**CDH2 (N-cadherin)**	TCGCTGCTTTCATACTGAACTTT	AGCGCAGTCTTACCGAAGG
	**TWIST1**	GGACAAGCTGAGCAAGATTCA	CGGAGAAGGCGTAGCTGAG
**Internal control genes**	**GOT1**	GCGCCTCCATCAGTCTTTG	ATTCATCTGTGCGGTACGCTC
	**HPRT**	TCAGTCAACGGGGGACATAAA	GGGGCTGTACTGCTTAACCAG
	**GAPDH**	AGGTCGGTGTGAACGGATTTG	TGTAGACCATGTAGTTGAGGTCA
